# Psychometric properties of the Swedish versions of Spinal Cord Independence Measure IV (SCIM IV) and Self-report (SCIM-SR) in inpatient and outpatient rehabilitation settings

**DOI:** 10.1038/s41393-026-01168-3

**Published:** 2026-01-22

**Authors:** Ulrica Antepohl, Emelie Butler Forslund, Peter Flank, Lisa Holmlund, Wolfram Antepohl, Richard Levi, STRIVE-SCI Consortium, Ulrica Antepohl, Emelie Butler Forslund, Peter Flank, Lisa Holmlund, Wolfram Antepohl, Richard Levi, Erik Berndtsson, Anna Granström, Tobias Holmlund, Xiaolei Hu, Claes Hultling, Jan Lexell, Maria Moschovou, Anna Olsson, Nora Sandholdt, Kristina Skill, Filip Tööj, Kerstin Wahman, Mikael Waller, Johanna Wangdell, Veronika Wiebols, Gunilla Åhrén, Elisabet Åkesson, Anestis Divanoglou, Sophie Jörgensen, Anestis Divanoglou, Sophie Jörgensen

**Affiliations:** 1 Clinical Department of Rehabilitation Medicine in Linköping, Region Östergötland, Linköping, Sweden; 2 Department of Health, Medicine, and Caring Sciences, Linköping University, Linköping, Sweden; 3 Department of Neurobiology, Care Science and Society, Division of Clinical Geriatrics, Karolinska Institutet, Huddinge, Sweden; 4 Department of Neurobiology, Care Science and Society, Division of Physiotherapy, Karolinska Institutet, Huddinge, Sweden; 5 R&D Unit, Aleris Rehab Station, Stockholm, Sweden; 6 Department of Community Medicine and Rehabilitation, Rehabilitation Medicine, Umeå University, Umeå, Sweden; 7 Department of Neurobiology, Care Sciences and Society, Division of Occupational Therapy, Karolinska Institutet, Stockholm, Sweden; 8 Department of Health Sciences, Lund University, Lund, Sweden; 9 Department of Rehabilitation Medicine, Skåne University Hospital, Lund, Sweden; 10 RG Aktiv Rehabilitering, Solna, Sweden; 11 Department of Rehabilitation Medicine, Ryhov Hospital, Jönköping, Sweden; 12 Department of Rehabilitation, Ängelholm Hospital, Ängelholm, Sweden; 13 Frykcenter Rehabilitering, Torsby, Sweden; 14 Department of Improvement and Innovation, Region Östergötland, Linköping, Sweden; 15 Gothenburg Competence Center for SCI, University of Gothenburg, Gothenburg, Sweden; 16 Department of Rehabilitation Medicine, Sunderby Hospital, Luleå, Sweden; 17 Department of Occupational Therapy and Physiotherapy, Sahlgrenska University Hospital, Mölndal, Sweden; 18 Department of Rehabilitation Medicine, Halland hospital, Halmstad, Sweden; 19 Swedish Association for Survivors of Accident and Injury (RTP), Sundbyberg, Sweden; 20 Department of Neurobiology, Care Science and Society, Division of Neurogeriatrics, Karolinska Institutet, Huddinge, Sweden; 21 R&D unit, Stockholms Sjukhem, Stockholm, Sweden

**Keywords:** Spinal cord diseases, Outcomes research

## Abstract

**Study design:**

Psychometric study.

**Objectives:**

To evaluate the data completeness, data distribution and ceiling/floor effects, internal consistency and convergent validity of the Swedish versions of the Spinal Cord Independence Measure IV (s-SCIM IV) and the Spinal Cord Independence Measure Self-report (s-SCIM-SR).

**Setting:**

Swedish inpatient and outpatient spinal cord injury (SCI) rehabilitation.

**Methods:**

The translation process was based on established guidelines with researchers, clinicians and consumers. s-SCIM IV and FIM^TM^ assessments were performed by observation and/or interview, s-SCIM-SR through self-report using paper forms.

**Results:**

In total, 101 participants (82% men) were included. There were no missing data for s-SCIM IV and 92% had answered all items in s-SCIM-SR. No ceiling or floor effects were observed. Cronbach´s alpha for the total s-SCIM IV scale was 0.91 (subscales 0.68–0.93) and for the total s-SCIM-SR scale 0.91 (subscales 0.62–0.93), with the lowest alphas for Respiration and Sphincter Management in both outcome measures. s-SCIM IV and s-SCIM-SR correlated strongly with each other and with FIM^TM^.

**Conclusions:**

Our results support the data completeness, lack of ceiling/floor effects, internal consistency (except the Respiration and Sphincter Management subscale) and convergent validity of the s-SCIM IV and s-SCIM-SR. Based on this initial psychometric testing, these outcome measures can be considered suitable to assess physical independence in inpatient and outpatient rehabilitation and long-term follow-up after SCI, for both clinical and research purposes. The now available Swedish versions of SCIM will enable a uniform national assessment of SCI-specific physical independence and facilitate research and international collaborations and comparisons.

## Introduction

To assess the outcome of interventions and to monitor improvements after spinal cord injury (SCI), valid and reliable injury-specific outcome measures are needed [1], in inpatient and community rehabilitation settings. Such outcome measures need to be translated, culturally adapted, and validated in different national contexts to facilitate both comparison and pooling of international data as well as to allow for multi-center studies [2].

The Spinal Cord Independence Measure (SCIM) was developed as a comprehensive clinician-administered assessment to evaluate the performance of daily activities in individuals with SCI [3]. It comprises three subscales: Self-care, Sphincter and Respiratory Management, and Mobility [3]. The total score ranges from 0–100 where higher scores indicate greater functional capacity and greater physical independence. The agreement with the generic Functional Independence Measure (FIM^TM^) [4] is high, but the SCIM has been found to be more sensitive to changes in function after SCI [5, 6]. SCIM has become a standard outcome measure in clinical practice and clinical studies worldwide [7].

The most recent version, SCIM IV [7] is a development of SCIM III. Despite its widespread use, studies have shown some psychometric limitations in the SCIM III, for example an impaired internal consistency in some items and low interrater reliability for some items [7]. In addition, the developers of SCIM III received feedback on content and phrasing of items from users of the assessment tool [7]. Thus, SCIM IV was developed based on the limitations found in the psychometric properties of SCIM III and feedback of staff members and international experts. SCIM IV was developed to focus on assessing specific patient conditions or situations that SCIM III did not address and thereby provide more accurate definitions of certain scoring alternatives [7]. SCIM IV has been shown to be valid and reliable in an international multi-center study with satisfying psychometric properties [7]. To enable people with SCI to evaluate their physical independence and facilitate data collection in community settings, a self-report version of SCIM III (SCIM-SR) has also been developed [8]. However, there is no self-report version explicitly corresponding to SCIM IV. As SCIM IV and SCIM-SR are being translated and used in different rehabilitation settings, further testing is needed.

Until recently, the lack of Swedish versions of internationally used and recommended SCI-specific outcome measures has limited their implementation in clinical practice, in research and in the Swedish quality registry for rehabilitation (SveReh). This limits the ability of Swedish rehabilitation professionals to assess independence in SCI-specific tasks and changes therein. As part of the research project Inter-PEER [9], the SCIM-SR was translated into Swedish (s-SCIM-SR) through a rigorous process to ensure accurate linguistic translation and cultural adaptation. Psychometric testing of s-SCIM-SR has been conducted in a community-based rehabilitation setting, showing sound psychometric properties [10]. We have now translated the clinician-administered SCIM IV into Swedish (s-SCIM IV) through a similar process. To our knowledge, there have been no studies testing a translated version of the SCIM IV, and no studies exploring correlations between SCIM IV and SCIM-SR.

Thus, the aim of the current study was to evaluate the psychometric properties (data completeness, data distribution and ceiling/floor effects, internal consistency reliability and convergent validity) of s-SCIM IV and s-SCIM-SR in inpatient and outpatient rehabilitation settings to ensure their usability along the entire hospital-based rehabilitation continuum.

## Methods

### Design

This cross-sectional study is part of the pilot phase of the research project STRIVE-SCI (Strengthening the rehabilitation continuum and optimizing the path to regaining a good life following acute SCI) in Sweden. STRIVE-SCI is a national, prospective, multi-center project aiming to generate comprehensive knowledge of patient outcomes, needs and experiences following an acute SCI in Sweden. The project protocol has been approved by the Swedish Ethical Review Authority (dnr 2022-01962-01). The pilot phase was conducted with the aims of testing study procedures and establishing the psychometric properties of the outcome measures that were recently translated into Swedish as part of Inter-PEER and STRIVE-SCI.

### Translation process of SCIM IV to s-SCIM IV

**Fig. 1:**
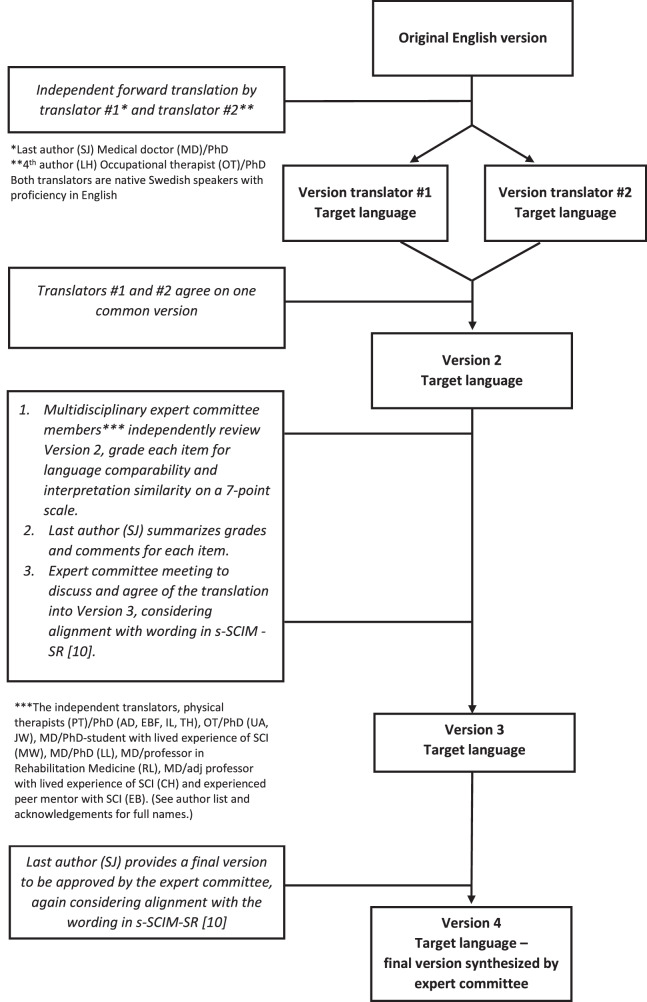
Translation process for s-SCIM IV. Flow chart of the translation process of SCIM IV to s-SCIM IV.

A flow chart of the translation process of SCIM IV to s-SCIM IV can be found in Fig. 1. Overall, the expert committee reached consensus on all translated items. Some items rendered discussions about scoring, and the developer was contacted for clarifications. One example was the item 6.6 (“Sufficient bladder emptying (PVR < 100cc) or intermittent self-catheterization; assistance for applying drainage instrument and/or insufficient ( < 300 cc) bladder filling”) where we found it unclear if “assistance for applying drainage instrument” referred to assistance with catheterization or assistance only for applying an external drainage instrument such as a condom catheter. The latter interpretation was supported by the developer, and this definition was clarified in the Swedish version. The developer also emphasized that observation is preferred, and assumptions should never be used. If observation is not possible, the rater should ask the person or the rehabilitation team about activity performance. In item 9 (Mobility in bed and action to prevent pressure sores), the wording “doing push-ups in the wheelchair” was culturally adapted to fit clinical practice in Sweden and thus replaced by the equivalent phrase “lifting oneself or leaning in the wheelchair for pressure relief”. In the Mobility subscale, the walking aid *“rollator”* (4-wheeled walker) was added as this is a very common aid in Sweden.

### Participants and procedures

Participants in the pilot phase were recruited between November 2022 and December 2023 from 10 clinics representing different regions in Sweden (Skåne, Stockholm, Västerbotten, Värmland, Halland, Östergötland, and Jönköping), providing inpatient and outpatient rehabilitation as well as lifelong follow-up. Three of these healthcare regions (Skåne, Stockholm, Västerbotten) also conduct the national highly specialized initial inpatient rehabilitation (Nationell högspecialiserad vård). Together the clinics represent both densely and sparsely populated geographical areas from the north to the south of Sweden. Inclusion criteria for the pilot phase were: (1) having a SCI (traumatic or non-traumatic); (2) aged ≥ 18 years; (3) adequate cognitive capacity to complete questionnaires, as determined by rehabilitation professionals based on clinical judgment and medical records; and (4) Swedish speaking. A study coordinator at each participating site approached eligible participants with information about the study and obtained written informed consent prior to inclusion. To promote uniform scoring and limit the risk of missing data, the study coordinator and clinicians involved in data collection had received instructions and participated in a workshop on how to administer and score s-SCIM IV. This workshop includes a newly developed instructional video presenting items where there is a need for clarifications and two study cases to exemplify the assessment of different levels of functioning. The video is openly available and has English subtitles (link provided at the end of the manuscript). Data were collected through self-report questionnaires, interviews, and observation. For each case, data were collected within 1–2 days, and the sequence of administering the outcome measures varied between clinics depending on local clinical routines. Information about which phase of rehabilitation the participant was undergoing was extracted from the medical record.

### Data collection

#### Sociodemographics and injury characteristics

Data on sex, age, education, employment status, marital status, date of injury, cause of injury, neurological level, and completeness according to the International Standards for Neurological Classification of SCI [11], were extracted from medical records and completed by self-report.

#### Spinal Cord Independence Measure (SCIM IV)

s-SCIM IV assessments were managed mainly by observation; if direct observation could not be accomplished (e.g., in outpatient settings), assessments were completed through interviews. SCIM IV consists of three subscales: the Self-care subscale comprises items 1–4 (0–20 points), the Respiration and Sphincter Management subscale comprises items 5–8 (0–40 points), and the Mobility subscale comprises items 9–14 (0–40 points). SCIM IV has been found to be valid, reliable and responsive, and can thus be used for clinical and research trials, including international multi-center studies. Scores on group level can be compared with those of SCIM III [7].

#### Spinal Cord Independence Measure Self-report (SCIM-SR)

SCIM-SR is based on SCIM III [8] and consists of the same three subscales as SCIM IV with the same subscale and total score ranges. Sound psychometric properties of the s-SCIM-SR in a community rehabilitation setting were found [10], corroborating results from other international studies [12, 13]. Prodinger et al. [1] used Rasch analysis to examine the internal construct validity and reliability of the SCIM-SR in a community survey and found that SCIM-SR violated certain assumptions of the Rasch measurement model. Prodinger et al. [1] therefore suggested an intermediate solution with anchoring sub-group characteristics on a common testlet and recommended the computation of Rasch transformed SCIM-SR scores. These transformations are only relevant for total scores and not for domain scores. As we are interested in both domain and total scores, we chose not to use that approach in the present study. Data for SCIM-SR were collected through self-reported completion of the questionnaire in paper format.

#### Other outcome measures

The generic FIM^TM^ [4] was used to assess the convergent validity of the s-SCIM IV and s-SCIM-SR. FIM^TM^ assessments were performed by observation and/or interview.

### Statistical analyses

#### Data completeness

To evaluate data completeness, the percentage of missing data for each item, each subscale and the total score was calculated for s-SCIM IV and s-SCIM-SR.

#### Data distribution and ceiling/floor testing

Score distributions and floor and ceiling effects were examined for s-SCIM IV and s-SCIM-SR. Floor and ceiling effects are present if more than 15% of participants achieve the lowest or highest score [14].

#### Internal consistency reliability

Internal consistency reliability was evaluated by using the Cronbach´s alpha coefficient for the full scale and each subscale for s-SCIM IV and s-SCIM-SR. Cronbach´s alpha between 0.70 and 0.95 for the scale indicates good internal consistency [14].

#### Validity

Convergent validity was evaluated by correlation analyses (Spearman’s rho; r_s_) between the different subscale scores in s-SCIM IV and s-SCIM-SR, and between the total score of s-SCIM IV, s-SCIM-SR, and FIM^TM^. The level of significance used was p < 0.05. For testing correlations of subscales in s-SCIM IV and s-SCIM-SR in relation to FIM^TM^, FIM^TM^ items A-M were divided into subscales matching those of SCIM; items A-E correspond to self-care, item F “Toileting” which according to FIM is part of self-care, was grouped with item G “Bladder” and H “Bowel” to better match the subscale Respiration and Sphincter Management in SCIM. All analyses were carried out using IBM Statistics SPSS version 29.

## Results

### Sociodemographic and injury characteristics

A large majority of the 101 participants were men (82%) and the median time since injury was 2 years, with a range of 0–54 years. Approximately 64% had been living with SCI for less than 5 years. A majority (66%) had a traumatic lesion, and 44% had AIS D. Table 1 presents the sociodemographics and injury characteristics. Participants underwent different phases of rehabilitation: 43% inpatient rehabilitation, 17% outpatient rehabilitation, 36% visit as part of lifelong follow-up, 5% research-specific visit.

**Table 1:** Sociodemographics and injury characteristics of the participants (n = 101).

Sex, n (%)	
Male	83 (82%)
Female	18 (18%)
Age, median (IQR) [min-max], years	62.0 (23) [22–86]
Time since injury, median (IQR) [min-max], n (%)	2.0 (8) [0–54]
<1 year	27 (27%)
1–4 years	38 (38%)
5–9 years	16 (16%)
10–19 years	9 (9%)
20–29 years	2 (2%)
30–39 years	4 (4%)
40–54 years	5 (5%)
Cause of injury, (missing = 1), n (%)	
Traumatic	66 (66%)
Non-traumatic	34 (34%)
Level and severity of injury^1^ (missing = 1), n (%)	
C1-4; AIS A, B, and C	25 (25%)
C5-8; AIS A, B, and C	6 (6%)
T1-S3; AIS A, B, and C	25 (25%)
AIS D at any neurological level of injury	44 (44%)
Main mode of mobility, n (%)	
Manual Wheelchair	67 (66%)
Power wheelchair	8 (8%)
Walking device (e.g. crutches, walking frame)	12 (12%)
No assistive devices	14 (14%)
Education, n (%)	
Elementary school (9 years)	18 (18%)
High school/Vocational training (12 years)	52 (52%)
University	28 (28%)
Other	3 (3%)
Living situation	
Single without children living at home	38 (38%)
Single with children living at home	4 (4%)
Married/cohabiting without children living at home	38 (38%)
Married/cohabiting with children living at home	16 (16%)
Living with parents	1 (1%)
Living with another close person	2 (2%)
Other	2 (2%)
Employment status^2^	
Employed	23 (20%)
Employed, on sick leave >3 months	14 (12%)
Self-employed	8 (7%)
Unpaid work (e.g. family business)	1 (1%)
Housewife/househusband	4 (3%)
Student	3 (3%)
Unemployed	1 (1%)
Retired due to health condition	25 (21%)
Retired due to age	38 (33%)

^1^The cohort did not include any participants with C1-C4 AIS A.

^2^Several options where possible to choose for each participant.

### The Swedish version of the Spinal Cord Independence Measure (s-SCIM IV)

#### Scoring

The mean (SD; min-max) total score was 54 (27; 0–100). Table 2 shows the total and subscale scores of SCIM IV and SCIM-SR.

**Table 2:** Total and subscale scores of s-SCIM IV and s-SCIM-SR.

	Total score (0–100)		Subscale Self-care (0–20)		Subscale Respiration & Sphincter Management (0–40)		Subscale Mobility (0–40)	
	s-SCIM IV	s-SCIM SR	s-SCIM IV	s-SCIM SR	s-SCIM IV	s-SCIM SR	s-SCIM IV	s-SCIM SR
Total, *n* (%)	101 (100)	93 (92)	101 (100)	101 (100)	101 (100)	96 (95)	101 (100)	98 (97)
Missing, *n* (%)	0 (0)	8 (8)	0 (0)	0 (0)	0 (0)	5 (5)	0 (0)	3 (3)
Mean (s.d.)	53.67 (26.9)	54.97 (26.2)	11.87 (6.6)	12.39 (6.9)	25.81 (10.4)	26.22 (9.7)	15.99 (12.6)	16.98 (12.4)
Median	59.0	59.0	14.0	15.0	27.0	27.0	15.0	16.0
IQR	43.5	44.0	12.5	13.0	18.0	18.0	18.0	18.2
Range scoring (min-max)	0–100	5–100	0–20	0–20	0–40	5–40	0–40	0–40

IQR: Inter-quartile range.

#### Data completeness

There were no missing data for s-SCIM IV.

#### Data distribution and ceiling/floor testing

The score covered the full possible range in both total score and all subscales (Table 2). Ratings of the lowest possible score were observed in both the total score (1%, n = 1) and the subscales; Self-care 4% (n = 4), Respiration and Sphincter Management 2% (n = 2), and Mobility 6% (n = 6). Ratings of the highest possible score were noted in all subscales; 9% (n = 9) for Self-care, 9% (n = 9) for Respiration and Sphincter Management and 8% (n = 8) for Mobility. One participant achieved the highest possible total score.

#### Internal consistency reliability

The Cronbach´s alpha coefficient for the full scale was 0.91, for Self-care 0.93, for Respiration and Sphincter Management 0.68, and for Mobility 0.92.

### The Swedish version of the Spinal Cord Independence Measure Self-report (s-SCIM-SR)

#### Scoring

The mean (SD; min-max) total score was 55 (26; 5–100), see Table 2.

#### Data completeness

Ninety-three participants (92%) had answered all items in the s-SCIM-SR. Some missing data were found in two of the three subscales, with response rates of 95% (Respiration and Sphincter) and 97% (Mobility).

#### Data distribution and ceiling/floor testing

**Fig. 2:**
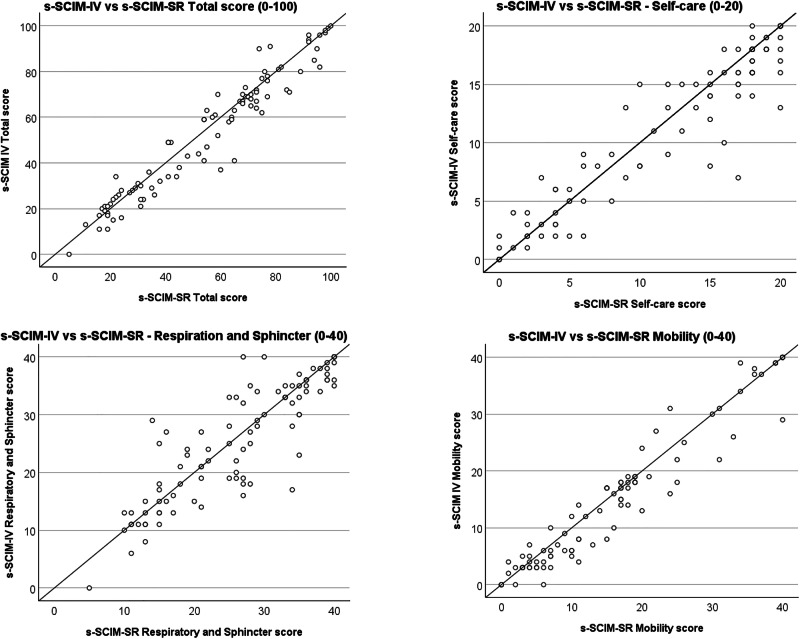
Distribution of SCIM scores. Graphs showing the distribution of s-SCIM IV and s-SCIM-SR domain and total scores.

Figure 2 shows the distribution of SCIM IV and SCIM-SR total and domain scores. The total score ranged from 5–100 (full range: 0–100), with full range in both Self-care (0–20) and Mobility (0–40), whereas Respiration and Sphincter Management ranged from 5–40 (full range: 0–40) (Table 2). Ratings of the lowest possible score were observed in Self-care 6% (n = 6) and Mobility 4% (n = 4). Ratings of the highest possible score were noted in all subscales; 13% (n = 13) for Self-care, 8% (n = 8) for Respiration and Sphincter Management and 9% (n = 9) for Mobility. One participant rated the highest possible score on the total score.

#### Internal consistency reliability

The Cronbach´s alpha coefficient for the full scale was 0.91, for Self-care 0.93, for Respiration and Sphincter Management 0.62 and for Mobility 0.90.

#### Validity for s-SCIM IV and s-SCIM-SR

There were strong positive correlations between the corresponding subscales in s-SCIM IV and s-SCIM-SR (Table 3); Self-care r_s_ = 0.93, p < 0.001, Respiration and Sphincter Management r_s_ = 0.89, p < 0.001, and Mobility r_s_ = 0.96, p < 0.001. Further, there was a strong positive correlation between the total score of s-SCIM IV and s-SCIM-SR (r_s_=0.97, p < 0.001).

**Table 3:** Correlations between s-SCIM IV, s-SCIM-SR and FIM, presented separately for total scores and the corresponding subscales.

Measure 1	Measure 2	Spearman’s rho	p-value
Total Score			
s-SCIM IV Total	FIM Total	0.901	<0.001
s-SCIM IV Total	s-SCIM-SR Total	0.967	<0.001
FIM Total	s-SCIM-SR Total	0.896	<0.001
Self-care			
s-SCIM IV Self-care	FIM A–E (Self-care)	0.917	<0.001
s-SCIM IV Self-care	s-SCIM-SR Self-care	0.930	<0.001
FIM A–E (Self-care)	s-SCIM-SR Self-care	0.907	<0.001
Respiration/Sphincter			
s-SCIM IV Resp & Sphincter	FIM F–H (Resp/Sphincter)	0.815	<0.001
s-SCIM IV Resp & Sphincter	s-SCIM-SR Resp & Sphincter	0.889	<0.001
FIM F–H (Resp/Sphincter)	s-SCIM-SR Resp & Sphincter	0.813	<0.001
Mobility			
s-SCIM IV Mobility	FIM I–M (Mobility)	0.944	<0.001
s-SCIM IV Mobility	s-SCIM-SR Mobility	0.963	<0.001
FIM I–M (Mobility)	s-SCIM-SR Mobility	0.935	<0.001

A strong positive correlation was also found between the FIM^TM^ total score and s-SCIM IV (r_s_ = 0.90, p < 0.001) and s-SCIM-SR (r_s_ = 0.90, p < 0.001) total scores (Table 3). Using FIM^TM^ subscale A-M, including self-care, bladder and bowel care together with mobility, and thus excluding five items for communication and social cognition, the correlation was similar (r_s_ = 0.90, p < 0.001 for s-SCIM IV and r_s_ = 0.89, p < 0.001 for s-SCIM-SR).

FIM^TM^ Self-care (items A-E), FIM^TM^ Sphincter control (items F-H) and FIM^TM^ Mobility (items I-M) were positively correlated with the corresponding subscales of s-SCIM IV and s-SCIM-SR (r_s_ = 0.81–0.94, p < 0.001).

## Discussion

This study examines the psychometric properties of the Swedish version of SCIM IV and SCIM-SR in inpatient and outpatient rehabilitation settings to ensure their usability along the entire hospital-based rehabilitation continuum. The translation and adaptation process of the English versions of the SCIM IV and SCIM-SR into Swedish by the expert committee resulted in minor clarifications and cultural adaptations (see Methods section), that were endorsed by the tool developer. There were few missing data and no observed ceiling or floor effects. The internal consistency was generally high, although the subscale Respiration and Sphincter management exhibited lower Cronbach’s alpha values compared to the other subscales. Expected positive correlations between the two translated scales as well as with the FIM^TM^, support the convergent validity of s-SCIM IV and s-SCIM-SR.

### Study participants and translation process

The sample size of 101 participants is greater or similar to that of previously published studies of the psychometric properties of SCIM III and SCIM-SR [12, 15, 16]. Participants in the present study represented males and females, a wide range of ages (22–86 years) and duration of SCI (0–54 years), traumatic and non-traumatic injuries, and most levels and severities of injury. This, in combination with including participants undergoing different types of rehabilitation, provided a solid ground for our analyses.

The translation process was performed according to established guidelines [17–19] and included persons with SCI, clinicians and researchers. Both for self-reported and clinician-administered outcome measures, the items need to be clear and easy to understand to minimize misinterpretations as well as respondent and clinician burden. During discussions in the expert committee, we identified a need for clarification on how to score some items and contacted the developer. After reaching consensus, we developed a workshop for clinicians on how to administer and score s-SCIM IV, following recommendations proposed by Liu et al. [20]. This workshop has been delivered on several occasions and is continuously being provided to clinicians using the outcome measure in their practice and/or for research purposes.

### Scoring, data completeness and data distribution and ceiling/floor testing

For s-SCIM IV, participants in the present study scored lower on all subscales compared to a Swedish sample of older adults with long-term SCI who were assessed using the s-SCIM III [21]. The sample in Waller et al. [21] consisted of more individuals with AIS D injuries, which likely explains the differences. Similar results emerged for s-SCIM-SR when comparing the present study sample with participants and peer mentors in a community-based rehabilitation programme [10]. As the sample in the present study had a median age of 62 years and almost half of them were undergoing inpatient rehabilitation, a greater level of independence in the community-based, younger (median age 41 years) sample would be expected. A comprehensive study by Catz et al. [7] demonstrated higher scores at discharge from inpatient rehabilitation compared to admission, which further supports this reasoning. For the subscale Respiration and Sphincter Management, the sample in the present study reported a comparable level of independence as the community sample in Jörgensen et al. [10]. Possibly, a high level of independence and/or functioning in respiration and sphincter management is reached during inpatient rehabilitation as these are focus areas during this rehabilitation phase. Moreover, the present study did not include persons with motor complete injuries, neurological level C1-C4 who generally have low scores on the subscale Respiration and Sphincter Management. Persons sustaining motor complete high-level injuries in Sweden are initially admitted to dedicated clinics. These clinics were not included in this study. We observed slightly higher scores (although not statistically confirmed) on the s-SCIM-SR as compared to the s-SCIM IV, throughout all subscales. Similar results were found by Fekete et al. [8] whereas Wang et al. [22] found the opposite. Nevertheless, these small differences are unlikely to affect the overall performance of the outcome measures.

There were no missing data for the s-SCIM IV, and we believe this might indicate that the data collectors did not have any major difficulties assessing the participants. Most data collectors had participated in the workshop, which might have reduced the risk of missing data and promoted uniform scoring. In line with previous studies, there were fewer missing data in professionally administered tools as compared to patient self-reported questionnaires [23]. Data collectors were instructed to carefully check the participants’ answers and approach them again if data were missing by mistake. Furthermore, based on lessons learned from Inter-PEER [9], data collectors offered guidance to respondents if they had difficulties understanding any item. This probably contributed to few missing data for the s-SCIM-SR. The missing data were primarily found in the subscale Respiration and Sphincter Management, corroborating previous results [8]. This might reflect privacy issues with sharing bowel and bladder functioning and management.

### Internal consistency reliability

All subscales, except Respiration and Sphincter Management, showed good internal consistency, corroborating previous results from Jörgensen et al. [10] and others [7]. The Cronbach’s alpha for the Respiration and Sphincter Management subscale in the present study is suboptimal, similar (0.68 vs 0.65) to the value obtained in a large international study conducted by the tool developers [7]. Furthermore, the lower internal consistency of this subscale was also found in different versions of the SCIM [7, 22, 24, 25]. One explanation may be that, apart from the correlation between items in a subscale, also the number of items affects Cronbach’s alpha [26]. The Respiration and Sphincter Management subscale includes fewer items than the other subscales which may contribute to the lower alpha value. The items in this subscale also assess separate areas (i.e., respiration, bowel and bladder management) that are grouped together and contain an assessment of both independence/activity performance and body functions (e.g., “Bowel movements at desired timing, without assistance; no mishaps”), which can all contribute to the low correlation between items.

### Validity

The self-report version and the clinician administered Swedish versions of SCIM were highly correlated, supporting the convergent validity. Similar results were obtained by the developer of SCIM-SR, although showing slightly lower correlation coefficients than in the present study [8]. Fekete et al. [8] used the Pearson correlation coefficient which might contribute to these small differences. We also found high correlations between SCIM and FIM^TM^, indicating that both scales can assess functional independence in persons with SCI, although the SCIM has been shown to be more responsive to changes in persons with SCI [24, 27]. Removing the items in FIM^TM^ related to communication and cognition did not change the strength of the correlation. A resulting weaker correlation would have been problematic as the items in FIM^TM^ explicitly assessing independence are expected to correlate highly with SCIM. There were also strong correlations between the different subscales of s-SCIM IV and s-SCIM-SR and the corresponding subscales of the FIM^TM^. Thus, our results further support the convergent validity of the s-SCIM IV and s-SCIM-SR.

### Strengths and limitations

The study has several strengths, such as a heterogeneous population, a large proportion of AIS D similar to contemporary epidemiological data in Sweden [28], a relatively large study sample, few missing data and a thorough translation procedure. The inclusion of ten SCI clinics for data collection is also a strength, as the study sample represents regional diversity in Sweden. Many persons collecting data could imply a limitation due to the risks of misinterpretations and differences in scoring. However, we believe that delivering the workshop on SCIM assessment has reduced such potential bias. Moreover, the professionals collecting data have much experience in assessing persons with SCI using FIM^TM^ and the strong correlation between FIM^TM^ and s-SCIM IV confirm the accuracy of the SCIM assessment. An observed limitation is that there were only 18% females in the study sample. About one third of those sustaining an SCI in Sweden are females [28] and a more even sex distribution would have been preferred for the psychometric testing. Individuals with the most severe injuries (motor-complete injuries, neurological level C1-C4) were not represented in our sample as these injuries occur less frequently and the clinics providing initial rehabilitation for these injuries did not participate. This could potentially influence the psychometric properties and reduce generalizability. However, participants demonstrated a wide range of independence levels, including very low total scores. This suggests that the outcome measures capture variation across the full continuum of observed independence. Nevertheless, future studies should aim for a more balanced sex distribution and inclusion of the most severe injuries to ensure that the psychometric properties of the measures are equally applicable to both males and females, and for all types of SCI. As this study was part of a pilot study for a large national multi-center study with a wide selection of assessment tools, more extensive psychometric testing with for example test-retest reliability was not feasible. However, as our initial psychometric testing support the data completeness, the lack of ceiling or floor effects, and as the internal consistency showed similar results to previous studies, we would not expect our Swedish version to be less stable over time or between raters as compared to the original English version. However, future studies could benefit from confirming these assumptions to further strengthen the incentive to implement SCIM IV in Swedish clinical practice.

## Conclusions

The results support the data completeness, the lack of ceiling or floor effects, internal consistency reliability (with an exception of the Respiration and Sphincter Management subscale), and convergent validity of s-SCIM IV and s-SCIM-SR. Based on this initial psychometric testing, these outcomes measures can be considered suitable to assess physical independence in inpatient and outpatient rehabilitation and long-term follow-up after SCI, for both clinical and research purposes. The similar results for s-SCIM-SR in hospital-based and community rehabilitation settings also support the usefulness of this outcome measure along the full rehabilitation continuum. The now available Swedish versions of SCIM will enable a uniform national assessment of SCI-specific physical independence and facilitate research and international collaborations and comparisons.

### Link to instructional video

https://vimeo.com/1063254800/22e85587e8

https://vimeo.com/1086462884/aa6aac79ab

## Data Availability

Data can be shared upon reasonable request to the corresponding author.
